# Reduction of slaughter age of Hanwoo steers by early genotyping based on meat yield index

**DOI:** 10.5713/ajas.19.0503

**Published:** 2019-10-21

**Authors:** Chang Dae Jeong, Mahfuzul Islam, Jong-Joo Kim, Yong-Il Cho, Sang-Suk Lee

**Affiliations:** 1 Ruminant Nutrition and Anaerobe Laboratory, Department of Animal Science and Technology, Sunchon National University, Suncheon 57922, Korea; 2 Department of Biotechnology, Yeungnam University, Gyeongsan 38541, Korea; 3 Animal Disease and Diagnostic Laboratory, Department of Animal Science and Technology, Sunchon National University, Suncheon 57922, Korea

**Keywords:** Body Weight, Carcass Weight, Genotyping, Growth Pattern, Meat Yield Index

## Abstract

**Objective:**

This study was conducted to determine early hereditary endowment to establish a short-term feeding program.

**Methods:**

Hanwoo steers (n = 140) were equally distributed into four groups (35/group) based on genetic meat yield index (MYI) viz. the greatest, great, low, and the lowest at Jukam Hanwoo farm, Goheung. All animals were fed in group pens (5 animals/pen) with similar feed depending on the growth stage. Rice straw was provided *ad libitum*, whereas concentrate was fed at 5.71 kg during the growing period (6 to 13 mo) and 9.4 kg during the fattening period (13 to 28 mo). Body weight (BW) was measured at two-month intervals, whereas carcass weight was determined at slaughtering at about 31 months of age. The Affymetrix Bovine Axiom Array 640K single nucleotide polymorphism (SNP) chip was used to determine the meat quantity-related gene in the blood.

**Results:**

After 6 months, the highest (p<0.05) BW was observed in the greatest MYI group (190.77 kg) and the lowest (p<0.05) in the lowest MYI group (173.51 kg). The great MYI group also showed significantly (p<0.05) higher BW than the lowest MYI group. After 16 and 24 months, the greatest MYI group had the highest BW gain (p<0.05) and were therefore slaughtered the earliest. Carcass weight was significantly (p<0.05) higher in the greatest and the great MYI groups followed by the low and the lowest MYI groups. Back-fat thickness in the greatest MYI group was highly correlated to carcass weight and marbling score. The SNP array analysis identified the carcass-weight related gene BTB-01280026 with an additive effect. The steers with the allele increasing carcass weight had heavier slaughter weight of about 12 kg.

**Conclusion:**

Genetic MYI is a potential tool for calf selection, which will reduce the slaughter age while simultaneously increasing carcass weight, back-fat thickness, and marbling score.

## INTRODUCTION

The prime goal of the beef cattle industry is to provide highly desirable beef for consumers in the most efficient manner. The Hanwoo cattle breed has been subjected to intensive selection for meat quality and production traits [1], and, thus, a significant genetic improvement has been obtained for carcass weight and eye muscle area [2]. Hanwoo beef is considered the most expensive yet high-quality beef in Korea. One of the reasons is that Hanwoo farmers fatten their steers up to 30 to 32 months of age to improve the marbling score. However, Hanwoo meat qualities such as sirloin size, back-fat thickness, marbling score, and carcass weight are not significantly different between the slaughtering ages of 27 and 31 months. Additionally, the Hanwoo farmers do not raise their cattle for over 30 months because of the increase in feed cost. Therefore, the appropriate selection of calves or cows at an early stage is very important to ensure economic benefit by decreasing slaughtering age while simultaneously increasing meat quality.

In recent years several studies have reported the develop ment of selection indexes to increase the efficiency of beef production. Production traits considered are weight of calf at weaning, post-weaning daily gain, age at slaughter after fattening, frequency of dystocia, milk yield, and body weight (BW) [3]. The choice of traits is different in each country owing to differences in biological, management, and economical production systems [4]. However, feed efficiency and growth rate generally have a strong association with the economics of gain. Heritability estimates for growth, based on live weight, weight gain, and carcass traits have been extensively reported in the literature as reviewed by Koots et al [5] and Rios-Utrera and Van-Vleck [6]. Genetic correlations between measures of growth or size at different ages are usually high. Selection for the rapid rate of gain in post-weaning feeding tests usually increases both birth weight and size at maturation. However, increases in birth weight also cause an increase in calving difficulties.

Recently, genome-wide association studies using high density single nucleotide polymorphism (SNP) chips have been used to detect the genetics of quantitative trait loci (QTL) in several domestic livestock species [7]. For instance, carcass traits such as carcass weight, eye muscle area, back-fat thickness, and marbling score are the critical quantitative traits affecting beef cattle production [1]. Thus, a genome-wide association study would help understand the molecular mechanisms that regulate beef production in Hanwoo cattle. Early assessment of carcass quality for a Hanwoo individual that is based on the genetics of the genotyped animal is desirable for farmers, because the information would assist in early selection or better feeding management practices. Therefore, the objective of this study was an early diagnosis of hereditary endowment, which will lead to the establishment of a decrease in the slaughtering age or carcass day based on the results of genetic predictions.

## MATERIALS AND METHODS

### Animals and phenotype data

A total of 140 Hanwoo steers were equally distributed into four treatments groups (35 in each group) at 6 months of age based on the genetic meat yield index (MYI) viz. the greatest, great, low, and the lowest group at the Jukam farm, Goheung, Jeonnam, Korea. The selection index (SI) and MYI were set up by Korean Institute for Animal Products Quality Evaluation, Sejong city and National Institute of Animal Science, Wanju, Chonbuk, Korea. The MYI was calculated as, MYI = 71.414–(0.625×backfat thickness [mm])+(0.130×loin eye muscle area [cm^2^])–(0.024×carcass weight [kg]). The SI of an individual was calculated as, SI = (1×EBV of carcass weight + 1×EBV of loin eye muscle area – 1×EBV of backfat thickness + 6×EBV of marbling score), in which EBV is standardized estimated breeding value for each trait. The breeding values were predicted with pedigree information and carcass records of genetical relatives by Korea Animal Improvement Association, Seoul, Korea. The SI of the greatest, great, low, and the lowest MYI groups were 12.04 to 9.33, 9.33 to 8.50, 8.50 to 8.07, and 8.07 to 2.91, respectively. The first day BW was also collected from the farm record and included into analysis.

The steers were treated according to the recommendations described in “The Guide for the Care and Use of Laboratory Animals,” published by the Institutional Animal Care and Use Committee (IACUC) of National Institute of Animal Science (2012-C-037) in Korea (IACUC APPROVAL NUMBER: SCNU-IACUC-2012-4). The experimental house was large enough to raise 140 steers at a time without any disturbances. The width×length of each pan was 5 m×10 m with feeder and waterer facilities. The experimental animals were fed in group pens (five animals/pen) with a similar amount of feed depending on the growth stage. Rice straw was provided *ad libitum*, whereas concentrate feed was fed at 5.71 kg during the growing period (6th to 13th months) and 9.4 kg during the fattening period (13th to 28th months). Feed ingredients (as-fed basis) and chemical composition (% of dry matter) of the experimental diets used in the *in vivo* experiment are presented in Table 1. The BW was measured every two months, whereas carcass weight was determined after slaughter at approximately the 31st month of age. Average daily gain (kg/d) was measured as, ([BW of day 720 – BW of day 1]/720). The steers were slaughtered at the municipal slaughterhouse following the normal commercial slaughterhouse procedure. After 24 h post-slaughter chilling, carcass weight was measured and evaluated the carcasses based on the official grader of carcass traits following the Korean carcass grading standard (National Livestock Cooperatives Federation) [8]. Correlation analysis of growth patterns and carcass characteristics of Hanwoo steers were calculated along with the MYI.

### Genotyping and single nucleotide polymorphisms quality control

Blood samples of 140 Hanwoo steers were collected for genotyping using Affymetrix Bovine Axiom Array 640k SNP chips. Quality controls were tested to screen available SNPs with PLINK version 1.07 [9], and the SNPs meeting the following criteria were removed: i) the frequency of the least genotype was less than ten, ii) the call rate was smaller than 95%, iii) p-value of the Hardy-Weinberg equilibrium test was less than 0.001, and iv) minor allele frequency was less than 5%.

### Statistical analysis

The phenotypic data were analyzed using the general linear model for randomized complete block design using SAS version 9.1 [10]. Genome-wide association analyses were performed according to the method described by Aulchenko et al [11]. At first, slaughter-year-season were fit as a fixed effect and age of month as a covariate, using the SAS general linear model procedure in SAS version 9.1. After that, the fixed or covariate having 0.1 statistical significance level was fit into a mixed model with a genome-relationship matrix (G matrix) [12], as the pedigree information of the 140 steers was limited. The G matrix was constructed using an R subroutine (version 2.15.0), and the residuals of each phenotype were obtained from the equation of mixed model using ASREML (version 3.0). Secondly, the residuals were regressed on each SNP using a simple linear regression model using PLINK version 1.07. For each individual, SNP genotypes for BB, BA, and AA were defined as −1, 0, and 1, respectively, such that allele substitution effect by replacing B with allele A was estimated for each SNP. To set threshold values of statistical significance, 0.1% point-wise p-value from the F distributions was applied for each SNP test.

## RESULTS

The BW change by growth stage and carcass characteristics of Hanwoo steers as influenced by meat yield are presented in Table 2. After 6 months, the highest BW was observed (p< 0.05) in the greatest MYI group (191 kg) and the lowest (p< 0.05) in the lowest MYI groups (174 kg). The great MYI group also showed significantly (p<0.05) higher BW (187 kg) than that in the lowest MYI group. After a 16-mo (480 days) growing period, a significant (p<0.05) weight pattern was observed in the Hanwoo steers as 453, 421, 405, and 385 kg in the greatest, great, low, and the lowest MYI groups, respectively. The same decreasing pattern from the greatest to the lowest MYI group of Hanwoo steers was observed in the 24-mo growing period (720 days) with a 70.72 kg difference between the greatest and the lowest MYI group. Based on the average daily weight gain, the BW at day 900 was expected to be the highest in the greatest MYI group (774 kg; p<0.05). Accordingly, at slaughter, the greatest MYI group had significantly (p<0.05) the highest carcass weight (461 kg), followed by great (448 kg), low (425 kg), and then the lowest MYI group (409 kg).

The Pearson correlation analyses of parameters obtained from each treatment are shown in Tables 3, 4, 5, and 6. The BW at the beginning of the experiment in the greatest MYI group was positively correlated to 180 days (p≤0.01) and back-fat thickness (p≤0.05). After 480 days, the BW was also positively correlated to 720 days’ BW (p≤0.01) and carcass weight (p≤0.01) with *r* = 0.574 and *r* = 0.655, respectively. In addition, BW at 720 days was positively correlated to carcass weight (p≤0.01) and eye muscle area (p≤0.05). Highly significant (p≤0.01) positive correlations were also observed between carcass weight and eye muscle area (*r* = 0.457), and between eye muscle area and marbling score (*r* = 0.473). In the great MYI group, positive correlations were observed in the 480 and 720-days’ BW (p≤0.01) and carcass weight and back-fat thickness (p≤0.01). A negative correlation was observed between carcass day and eye muscle area (cm^2^).

The correlation analysis of growth pattern and carcass characteristics of the low MYI group showed that the BW at 180 days was positively correlated to the BW at 480 days (p≤ 0.05); however, it was negatively correlated to carcass weight (p≤0.05). A highly significant (p≤0.01) positive correlation was observed between the BWs at 480 and 720 days (*r* = 0.657). The carcass weight was also positively correlated to eye muscle area (cm^2^) (p≤0.01), back-fat thickness (p≤0.05) and marbling score (p≤0.05). Eye muscle area (cm^2^) was also positively correlated to marbling score (p≤0.01) with *r* = 0.501. At the beginning of the experiment, the BW of the lowest MYI group was positively correlated (*r* = 0.419) to the marbling score (p≤0.05). It was also observed that the BWs at 480 and 720 days were positively correlated (p≤0.01) to the BW at 720 days (*r* = 0.713) and the carcass weight (*r* = 0.515), respectively. The carcass weight was also positively correlated to eye muscle area.

For carcass traits, twenty-three (23) SNPs were detected (Tables 7, 8) that were located on *Bos taurus* autosomes (BTAs) 1, 2, 3, 4, 5, 6, 7, 11, 14, 16, 21, and 28. Among the 23 SNPs, only BTB-1280026 SNP was significantly (p<0.01) related to carcass weight with an additive effect of 12.4 kg for increasing C alleles over T alleles. However, for eye muscle area, back-fat thickness, and marbling score, there was limited statistical support for the association of the SNPs (Tables 7, 8).

## DISCUSSION

In the literature, the relationship between growth, feed efficiency, and carcass composition is reported as mainly dependent on feed management in terms of the composition of the diet or the amount of food (restricted versus *ad libitum*) [13–16]. Breed type, age, and weight at slaughter also influence the relationship between the traits [14,15,17]. However, in the present study, rice straw was given *ad libitum*, whereas concentrate feed was fed at 5.71 kg during the growing period (6 to 13 mo) and 9.4 kg during the middle fattening period (13 to 28 mo) to minimize the effect of the feed on BW gain and carcass weight.

In this experiment, the live and carcass weight, eye muscle area, and back-fat thickness was observed to be the highest in the greatest MYI group. This greatest MYI group had a significant (p≤0.01) positive correlation with live and carcass weight indicating a strong genetic correlation between live growth with carcass weight. There was also a significant correlation found in the greatest MYI group between carcass weight and eye muscle area as well as between eye muscle area and marbling score indicating the meat quality trait was also influenced by the greatest genotyped animals. This finding is consistent with that of Johnson et al [18], who reported a high and positive (0.64 to 0.97) genetic and phenotypic correlation between steer live weight and hot carcass weight. The genetic correlation between live animals and carcasses were also consistent with the reports for other beef cattle breeds [19–21]. A significant reduction in carcass day was also recorded in the greatest MYI group, which is expected by farmers. Lowering the carcass day has a positive impact on the reduction of total feed cost as steers slaughtered at the earliest age consume less feed overall [22]. In Table 2, eye muscle area (cm^2^) was decreasing linearly from the greatest to lowest group however in correlation analysis of the Great MYI group showed a significant negative correlation between carcass day and eye muscle area which was unexpected. This is may be due to their transfer to slaughterhouse at last as a result they received much more psychological stress which affects feed consumption and subsequently carcass quality and quantity [23].

Among the identified 23 SNPs, only the BTB-1280026 SNP was significantly associated with carcass weight. The number of steers for meat quality analysis in this study was very limited (n = 140), which may not enable detection of many SNPs for carcass quality traits with strong statistical evidence. As the sample size is one of the major factors for SNP detection in genome-wide association analysis [24], more samples are needed to detect QTL with strong statistical support, and the detected QTL (SNP) subsequently need to be validated by the addition of more samples. However, this study was in partial agreement with that of Lu et al [25], who found eight SNPs that were associated with hot carcass weight in 747 genotyped animals and 7 of which were located on BTA6. However, 520 SNPs were found significantly associated with mostly individual traits (473 SNPs), and multiple traits (47 SNPs) based on less stringent significance level (p<0.001) and 22 of SNPs out of 48, located on BTA6, were associated with hot carcass weight. Likewise, Edea et al [7] identified 17 and 16 QTLs that were significantly (p<0.01) associated with carcass weight under the additive and dominant models, respectively. They also mentioned that BTA 2, 6, 14, 22, and 24 loci were previously identified in several beef cattle breeds as QTL for carcass weight. In this study, BTB-1280026, the identified significant carcass weight-related SNP, was located on BTA 14, which was strongly supported by the results of Rempel et al [26], who identified carcass weight-related QTL (21.1 to 21.1 Mb) on BTA 14 in crossbred beef cattle. In addition, the QTLs on BTA 14 are also highly associated with body size in taurine and zebu cattle [27].

## CONCLUSION

The genetic MYI for calf selection is an important factor in determining beef quantity. Our results show that the use of the genetic MYI could reduce the slaughter age, while simultaneously increasing carcass weight as well as back-fat thickness and marbling score. High-density bovine SNP array analysis showed that one SNP, BTB-01280026, was found for carcass weight with strong statistical support, and the steers with the allele for increasing carcass weight had slaughter weight that was greater by about 12 kg than the animals with an alternate decreasing allele.

## Figures and Tables

**Table 1:** Ingredients and chemical compositions of feeds used for *in vivo* experiment

Parameters	Growing feed 6 to 13 mo	Fattening feed 13 to 28 mo	Rice straw1)
Ingredients of concentrate feed (as-fed basis)			
Corn	23.36	22.30	-
Wheat bran	12.00	12.00	-
Wheat flour	-	1.00	-
Wheat grain	11.82	5.06	-
Corn gluten feed	14.00	9.70	-
Molasses	6.00	6.00	-
Rapeseed meal	9.00	5.00	-
DDGS	-	4.00	-
Coconut Kernel Meal	3.50	5.50	-
Palm kernel meal	14.00	14.00	-
Tapioca	2.92	12.50	-
Salt dehydrated	0.60	0.60	-
Limestone (1 mm)	2.25	2.04	-
Vitamin premix2)	0.30	0.20	-
Mineral premix3)	0.25	0.10	-
Chemical Compositions (% of DM)			
DM	87.02	86.86	86.76
CP	14.00	13.99	6.41
EE	2.50	3.39	1.01
Ash	12.00	6.21	11.84
CF	18.00	8.93	32.24
NDF	37.00	39.29	86.50
ADF	15.00	17.04	55.20
TDN	69.23	74.11	49.80

mo, months; DDGS, distillers dried grains with solubles; DM, dry matter; CP, crude protein; EE, ether extract; CF, crude fiber; NDF, neutral detergent fiber; ADF, acid detergent fiber, TDN, total digestible nutrient.

1) Rice straw was provided *ad libitum* throughout the experiment.

2) Vitamin premix contained the following amount which was diluted in cellulose (g/kg premix): L-ascorbic acid, 121.2; DL-α-tocopherol acetate, 18.8; thiamin hydrochloride, 2.7; riboflavin, 9.1; pyridoxine hydrochloride, 1.8; niacin, 36.4; Ca-D-pantothenate, 12.7; myo-inositol, 181.8; D-biotin, 0.27; folic acid, 0.68; p-aminobenzoic acid, 18.2; menadione, 1.8; retinal acetate, 0.73; cholecalciferol, 0.003; cyanocobalamin, 0.003.

3) Mineral premix contained the following ingredients (g/kg premix): Mg SO_4_·7H_2_O, 80.0; NaH_2_PO_4_. 2H_2_O, 370.0; KCL, 130.0; Ferric citrate, 40.0; ZnSO_4_·7H_2_O, 20.0; Ca-lactate, 356.5; CuCl, 0.2; AlCl_3_·6H_2_O, 0.15; KI, 0.15; Na_2_Se_2_O_3_, 0.01; MnSO_4_·H_2_O, 2.0; CoCl_2_·6H_2_O, 1.0.

**Table 2:** Body weight change by growth stage and carcass characteristics of Hanwoo steers (mean±SE) as influenced by meat yield

Parameters	Treatment groups				p-value		
	
	Greatest MYI	Great MYI	Low MYI	Lowest MYI	Treat	L	Q
First day weight (kg)1)	25.09±0.56	25.40±0.54	24.83±0.51	24.60±0.64	ns	ns	ns
180 day weight (kg)	190.77±2.55^a^	186.51±3.55^ab^	178.26±3.49^bc^	173.51±4.04^c^	**	**	ns
480 day weight (kg)	452.74±3.78^a^	420.57±3.07^b^	404.66±5.82^c^	384.63±4.31^d^	**	**	ns
720 day weight (kg)	643.97±6.78^a^	617.74±4.21^b^	589.86±5.18^c^	573.26±6.43^d^	**	**	ns
Average daily gain (kg/d)	0.86±0.01^a^	0.82±0.01^b^	0.78±0.01^c^	0.76±0.01^d^	**	**	ns
Expected live weight at 900 d (kg)	773.61±8.44^a^	740.43±5.35^b^	706.29±6.45^c^	685.82±7.92^d^	**	**	ns
Carcass day	908.45±3.22	923.60±3.92	918.72±6.45	921.42±7.92	-	-	-
Carcass weight (kg)	461.46±4.98^a^	447.83±4.12^a^	425.07±6.17^b^	409.23±5.83^c^	**	**	ns
Eye muscle area (cm^2^)	93.12±1.36^a^	90.94±1.36^b^	88.97±2.09^c^	88.81±1.42^d^	ns	*	ns
Back-fat thickness (cm^2^)	12.03±0.75	12.14±0.71	12.76±0.86	11.90±0.72	ns	ns	ns
Marbling score	5.76±0.30	5.94±0.21	6.24±0.25	5.48±0.26	ns	ns	ns

SE, standard error; MYI, meat yield index; Treat, treatment effect; L, linear effect; Q, quadratic effect.

1) First day or birth weight (kg) was received from the farm records and considered as initial body weight for the analysis.

a–d Means in the same row with different superscript letters differ significantly (p<0.05).

Level of significance indicated by

* p<0.05,

** p<0.01,

ns, not significant.

**Table 3:** Correlation analysis of growth pattern and carcass characteristics of the greatest meat yield group

Parameters	First day	180 day	480 day	720 day	Carcass weight	Carcass day	Eye muscle area	Back fat thickness	Marbling score
First day	1.000	0.592**	−0.233	0.096	−0.104	0.302	−0.269	0.416*	−0.297
180 day	-	1.000	−0.272	0.002	−0.122	0.309	−0.261	0.224	−0.144
480 day	-	-	1.000	0.574**	0.655**	−0.185	0.279	0.042	0.262
720 day	-	-	-	1.000	0.652**	−0.340	0.410*	0.308	0.161
Carcass weight	-	-	-	-	1.000	0.026	0.457**	0.095	0.220
Carcass day	-	-	-	-	-	1.000	0.015	−0.047	−0.270
Eye muscle area	-	-	-	-	-	-	1.000	−0.108	0.473**
Back-fat thickness	-	-	-	-	-	-	-	1.000	−0.254
Marbling score	-	-	-	-	-	-	-	-	1.000

** Values are significantly different (p≤0.01).

* Values are significantly different (p≤0.05).

**Table 4:** Correlation analysis of growth pattern and carcass characteristics of great meat yield group

Parameters	First day	180 day	480 day	720 day	Carcass weight	Carcass day	Eye muscle area	Back fat thickness	Marbling score
First day	1.000	0.090	0.008	−0.071	−0.296	−0.121	0.055	−0.167	−0.178
180 day	-	1.000	−0.066	−0.280	−0.273	0.004	−0.219	0.097	−0.006
480 day	-	-	1.000	0.748**	0.176	−0.145	−0.190	−0.030	0.166
720 day	-	-	-	1.000	0.242	−0.103	−0.034	0.113	0.245
Carcass weight	-	-	-	-	1.000	0.120	−0.087	0.481**	0.104
Carcass day	-	-	-	-	-	1.000	−0.460**	0.068	−0.234
Eye muscle area	-	-	-	-	-	-	1.000	−0.119	0.231
Back-fat thickness	-	-	-	-	-	-	-	1.000	0.291
Marbling score	-	-	-	-	-	-	-	-	1.000

** Values are significantly different (p≤0.01).

* Values are significantly different (p≤0.05).

**Table 5:** Correlation analysis of growth pattern and carcass characteristics of low meat yield group

Parameters	First day	180 day	480 day	720 day	Carcass weight	Carcass day	Eye muscle area	Back fat thickness	Marbling score
First day	1.000	0.229	0.221	0.100	−0.066	0.083	−0.120	−0.101	0.213
180 day	-	1.000	0.368*	0.315	−0.372*	0.112	−0.342	0.070	0.135
480 day	-	-	1.000	0.657**	−0.307	0.168	−0.258	−0.197	−0.103
720 day	-	-	-	1.000	−0.287	−0.012	−0.236	−0.210	−0.209
Carcass weight	-	-	-	-	1.000	−0.031	0.623**	0.382*	0.389*
Carcass day	-	-	-	-	-	1.000	−0.246	0.039	0.116
Eye muscle area	-	-	-	-	-	-	1.000	0.005	0.501**
Back-fat thickness	-	-	-	-	-	-	-	1.000	0.027
Marbling score	-	-	-	-	-	-	-	-	1.000

** Values are significantly different (p≤0.01).

* Values are significantly different (p≤0.05).

**Table 6:** Correlation analysis of growth pattern and carcass characteristics of the lowest meat yield group

Parameters	First day	180 day	480 day	720 day	Carcass weight	Carcass day	Eye muscle area	Back fat thickness	Marbling score
First day	1.000	0.156	0.232	0.186	−0.126	−0.252	−0.110	0.049	0.419*
180 day	-	1.000	0.058	0.056	−0.148	−0.009	−0.197	0.175	−0.293
480 day	-	-	1.000	0.713**	0.177	0.348	−0.109	−0.175	0.030
720 day	-	-	-	1.000	0.515**	0.244	0.116	0.120	0.104
Carcass weight	-	-	-	-	1.000	0.247	0.560**	0.330	0.105
Carcass day	-	-	-	-	-	1.000	0.156	−0.258	−0.178
Eye muscle area	-	-	-	-	-	-	1.000	0.303	0.264
Back-fat thickness	-	-	-	-	-	-	-	1.000	0.175
Marbling score	-	-	-	-	-	-	-	-	1.000

** Values are significantly different (p≤0.01).

* Values are significantly different (p≤0.05).

**Table 7:** Identities, positions, and effect of the SNPs on carcass weight and eye muscle area of Hanwoo steers

SNP marker1)	SNP2)	BTA	Position3)	Additive4)	SEM	p-value
Carcass weight						
BTB-00986847	A/C	7	1,126,787	−6.39	2.50	0.106
NGS-21607	C/T	11	100,481,883	−9.15	3.12	0.035
BTB-1280026*	C/T	14	25,170,557	12.44	2.90	0.001
NGS-114518	A/G	28	40,462,756	−4.16	1.92	0.306
BTB-00995659	A/G	28	45,898,513	−9.19	3.95	0.204
Eye muscle area						
BTA-157501	C/T	3	76,469,248	1.11	0.37	0.027
NGS-28660	C/T	7	4,130,299	−1.41	0.45	0.019
BTB-01280026	C/T	14	25,170,557	1.58	0.68	0.208
BTA-00652140	C/T	16	53,663,332	−1.82	0.59	0.022
BTB-01107683	C/T	21	29,363,186	−1.41	0.52	0.066
NGS-114513	A/G	28	40,462,756	−1.32	0.46	0.039

SNP, single nucleotide polymorphism; BTA, *Bos taurus* autosome; SEM, standard error of the mean.

1,3) SNP marker annotations and their positions.

2) Alternative nucleotides, A/B alleles such that the estimated additive effect is for allele, A that is replaced with allele, B.

4) Estimate of additive effect of the SNP.

* Significant SNP marker is indicated by p<0.01.

**Table 8:** Identities, positions, and effect of the SNPs on back fat thickness and marbling score of Hanwoo steers

SNP marker1)	SNP2)	BTA	Position3)	Additive4)	SEM	p-value
Back-fat thickness						
NGS-26771	A/G	1	69,528,623	1.07	0.41	0.088
BTA-157501	C/T	3	76,469,248	−0.45	0.19	0.173
NGS-41558	A/C	3	123,148,964	−0.77	0.29	0.083
BTA-160954	A/G	5	18,775,860	−0.55	0.23	0.017
BTB-01312166	C/T	6	65,708,017	−1.17	0.49	0.017
BTB-00986847	A/C	7	1,126,787	−0.78	0.35	0.027
Marbling score						
BTA-90292	C/T	2	90,639,723	0.46	0.17	0.163
BTA-104512	A/G	4	112,700,199	−0.3	0.09	0.013
BTB-160954	A/G	5	18,775,860	−0.26	0.09	0.061
NGS-8401	G/T	5	76,428,730	−0.17	0.08	0.384
NGS-43407	A/G	5	110,911,478	0.22	0.08	0.045
BTB-00986847	A/C	7	1,126,787	0.29	0.12	0.039

SNP, single nucleotide polymorphism; BTA, *Bos taurus* autosome; SEM, standard error of the means.

1,3) SNP marker annotations and their positions.

2) Alternative nucleotides, A/B alleles such that the estimated additive effect is for allele, A that is replaced with allele, B.

4) Estimate of additive effect of the SNP.
